# Supplementary figures and images for: SES1 is vital for seedling establishment and post-germination growth under high-potassium stress conditions in Arabidopsis thaliana
Source: PeerJ. 2022 Oct 31;10:e14282. doi: 10.7717/peerj.14282 (PMC9632470; doi:10.7717/peerj.14282)

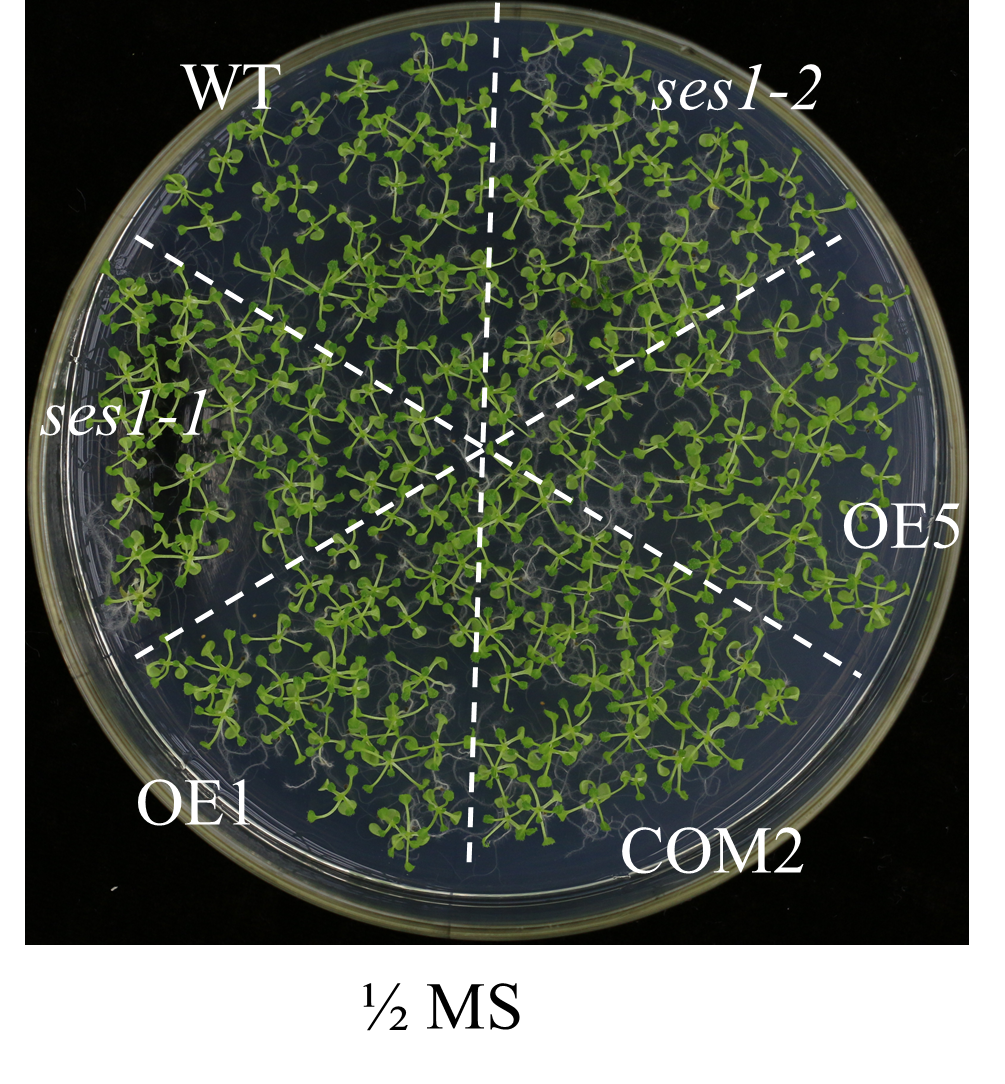

Supplement: Supplemental Information 1 [file peerj-10-14282-s001.zip › raw data1/Figure 1/Figure 1A/0.5MS-.png]

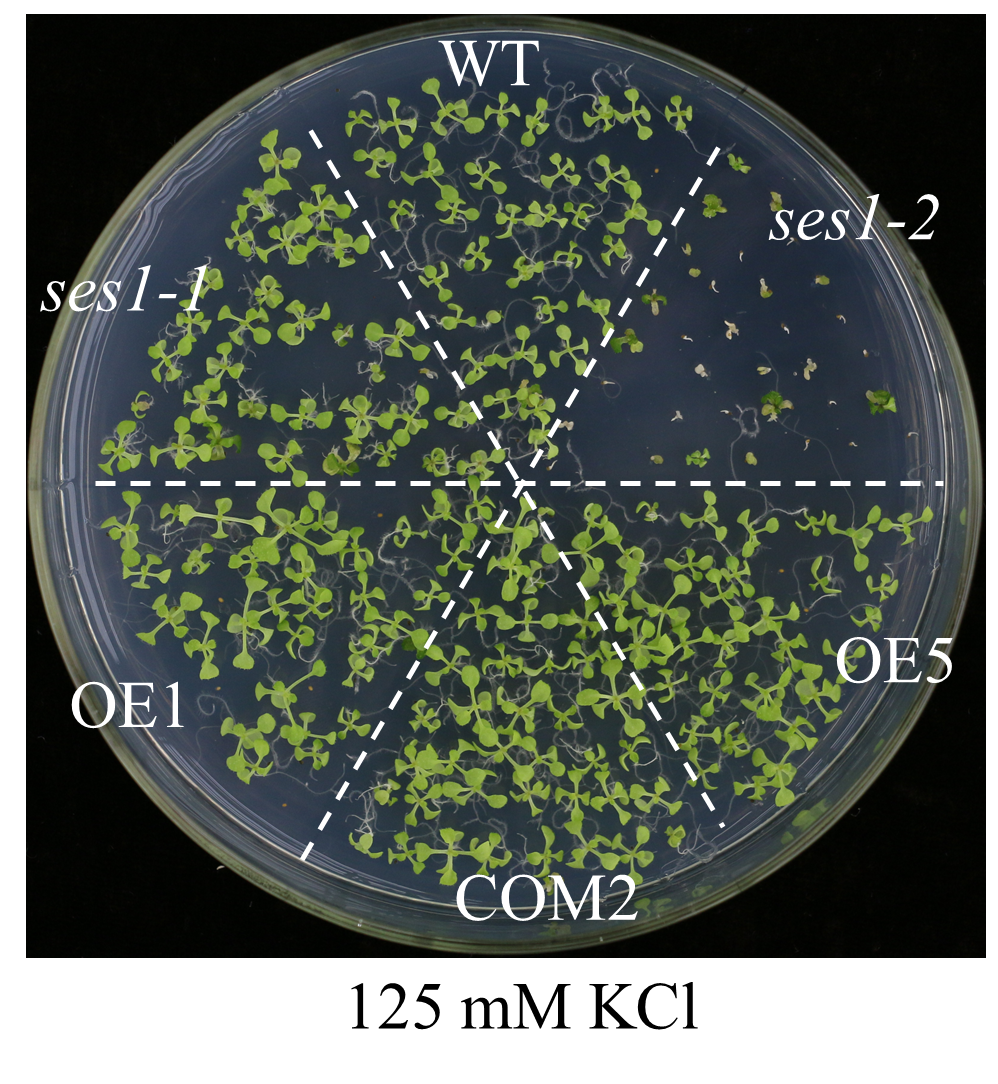

Supplement: Supplemental Information 1 [file peerj-10-14282-s001.zip › raw data1/Figure 1/Figure 1A/125mM KCl.png]

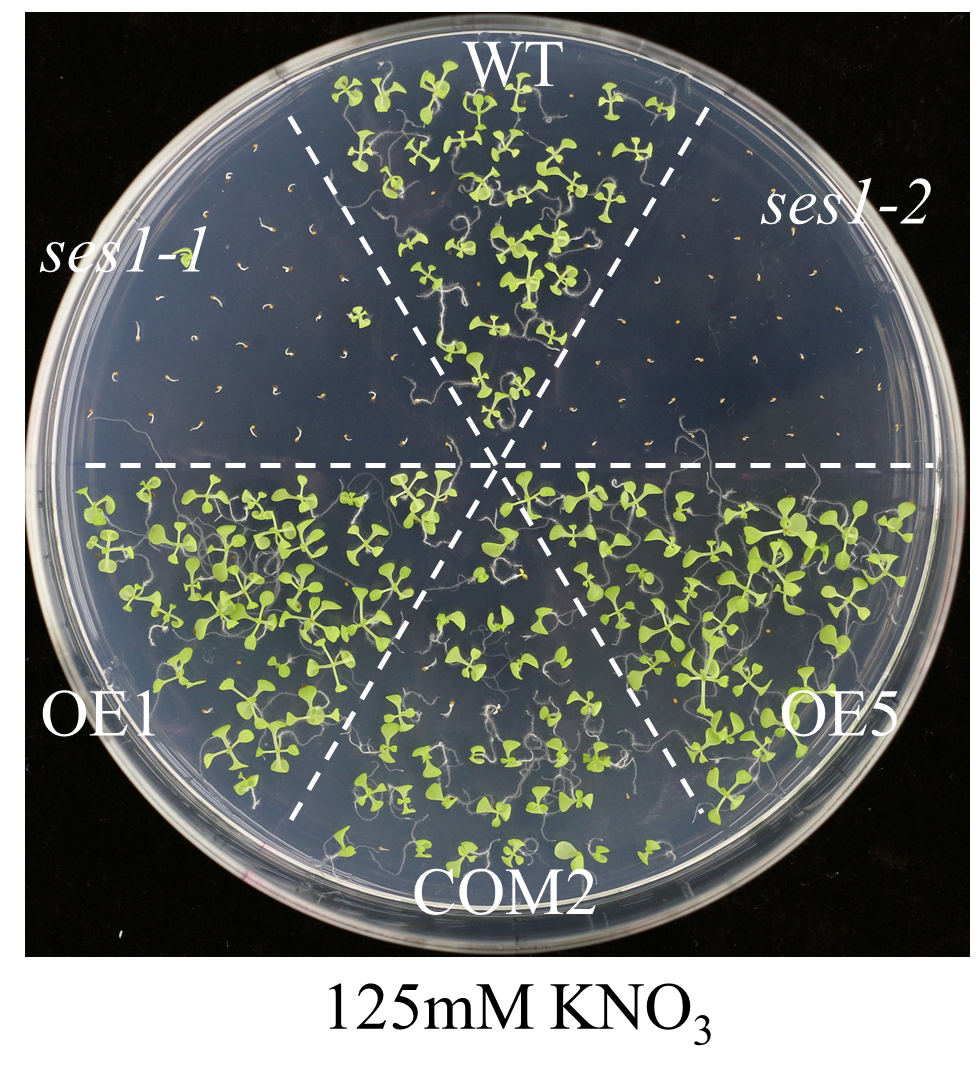

Supplement: Supplemental Information 1 [file peerj-10-14282-s001.zip › raw data1/Figure 1/Figure 1A/125mM KNO3.png]

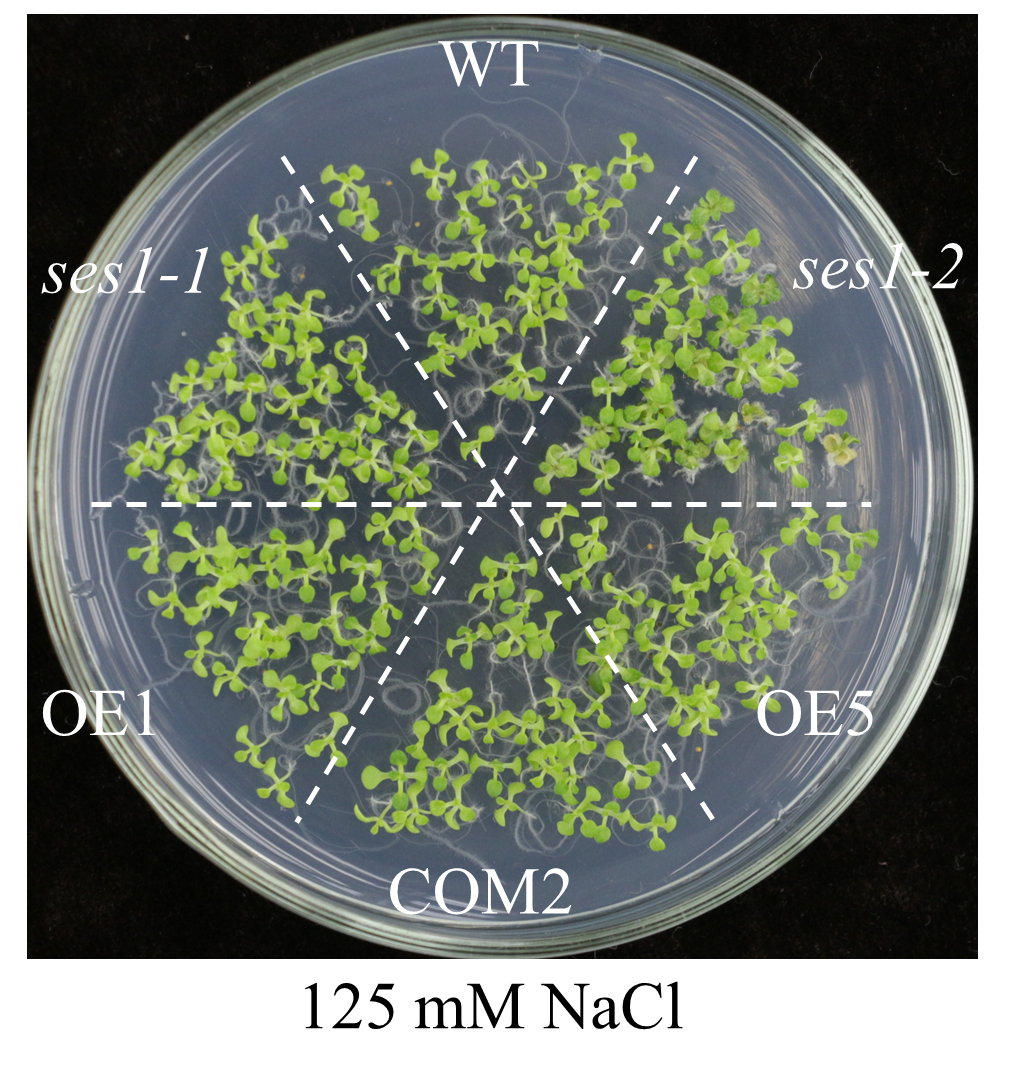

Supplement: Supplemental Information 1 [file peerj-10-14282-s001.zip › raw data1/Figure 1/Figure 1A/125mM NaCl.png]

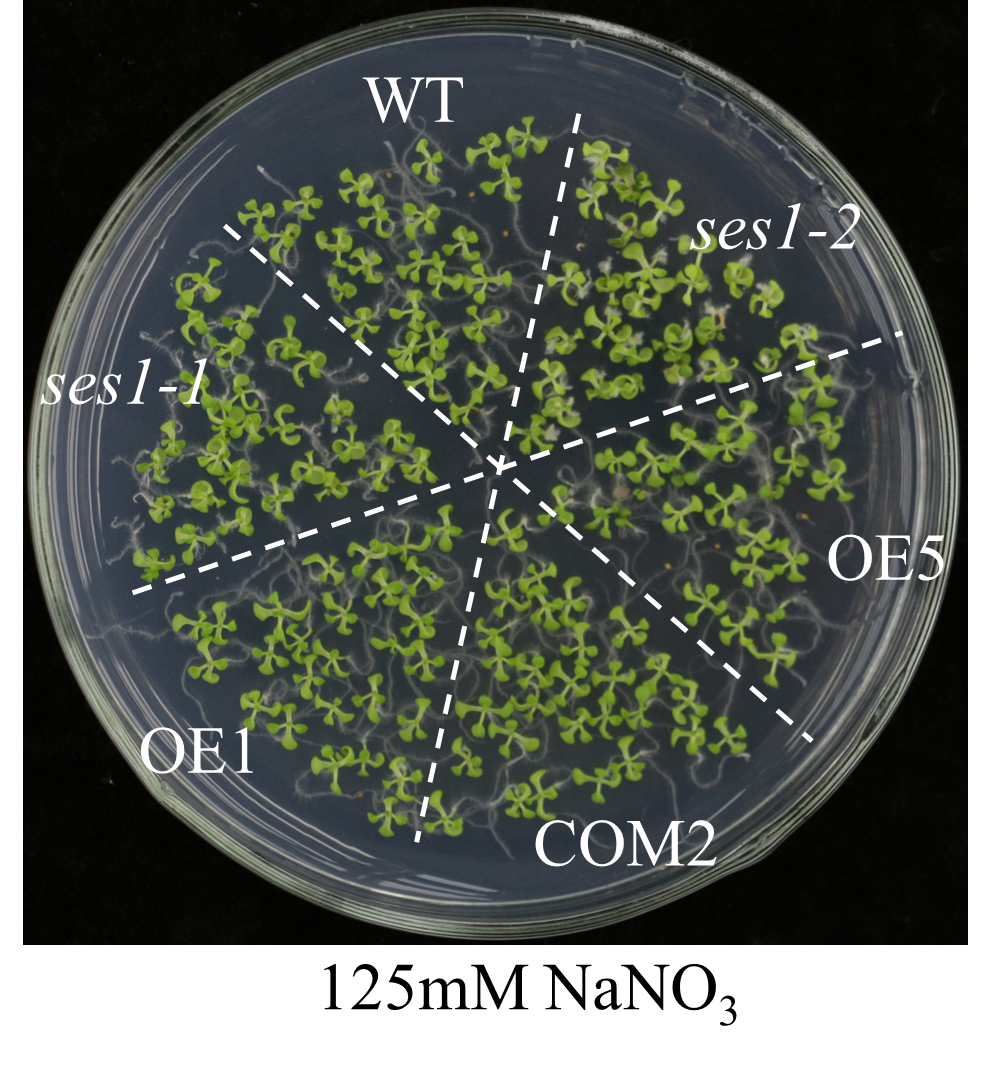

Supplement: Supplemental Information 1 [file peerj-10-14282-s001.zip › raw data1/Figure 1/Figure 1A/125mM NaNO3.png]

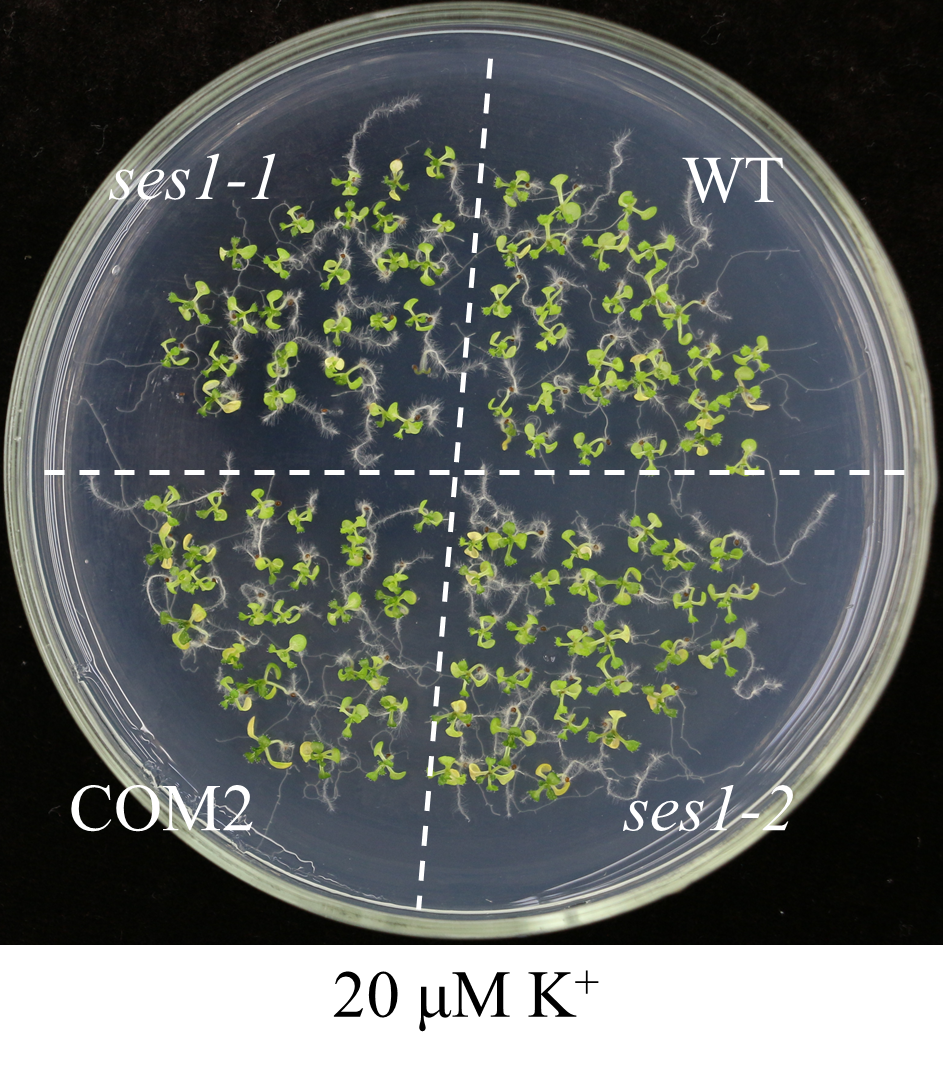

Supplement: Supplemental Information 1 [file peerj-10-14282-s001.zip › raw data1/Figure 1/Figure 1A/20μM K+.png]

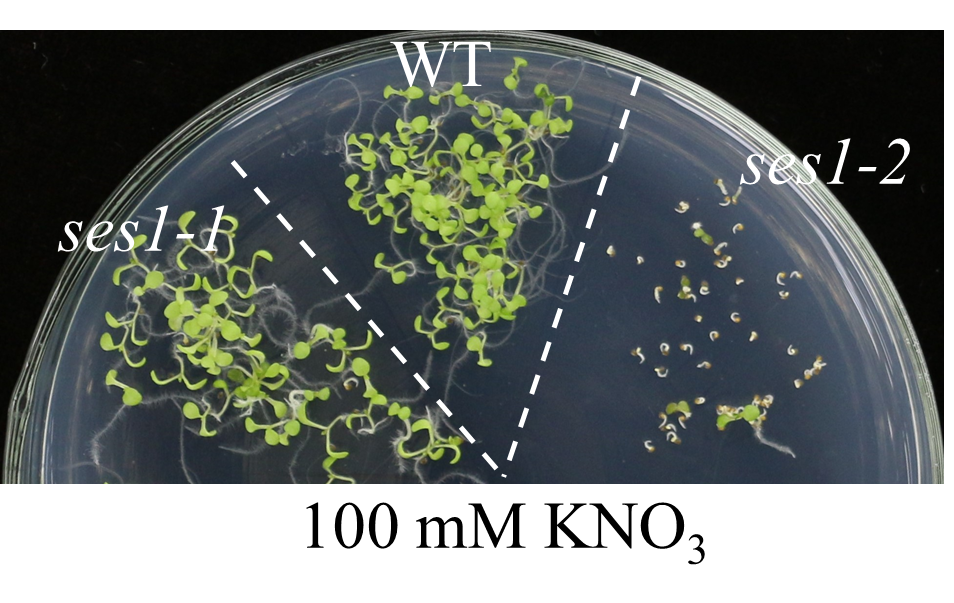

Supplement: Supplemental Information 1 [file peerj-10-14282-s001.zip › raw data1/Figure 2/Figure 2A/100mM KNO3.png]

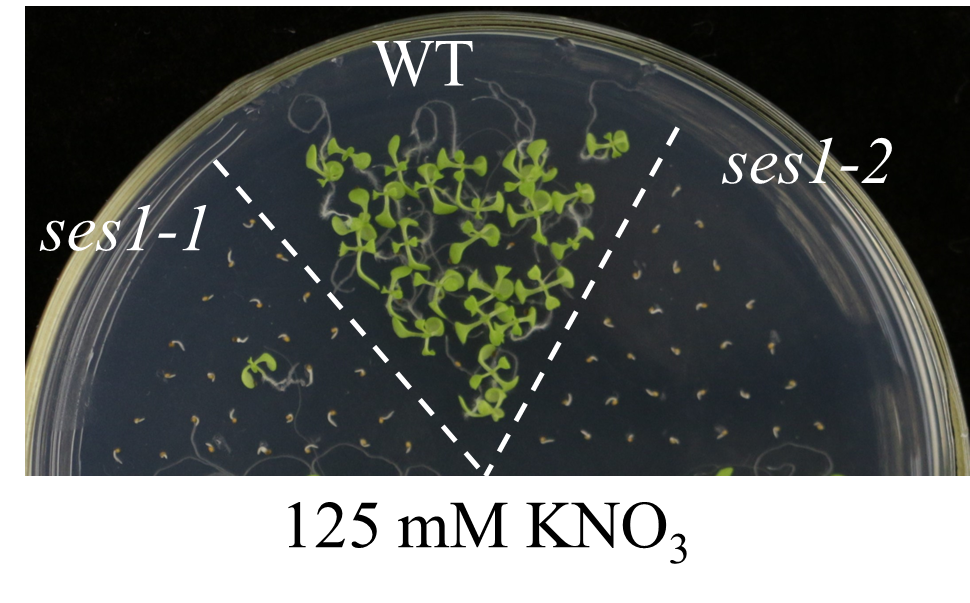

Supplement: Supplemental Information 1 [file peerj-10-14282-s001.zip › raw data1/Figure 2/Figure 2A/125mM KNO3.png]

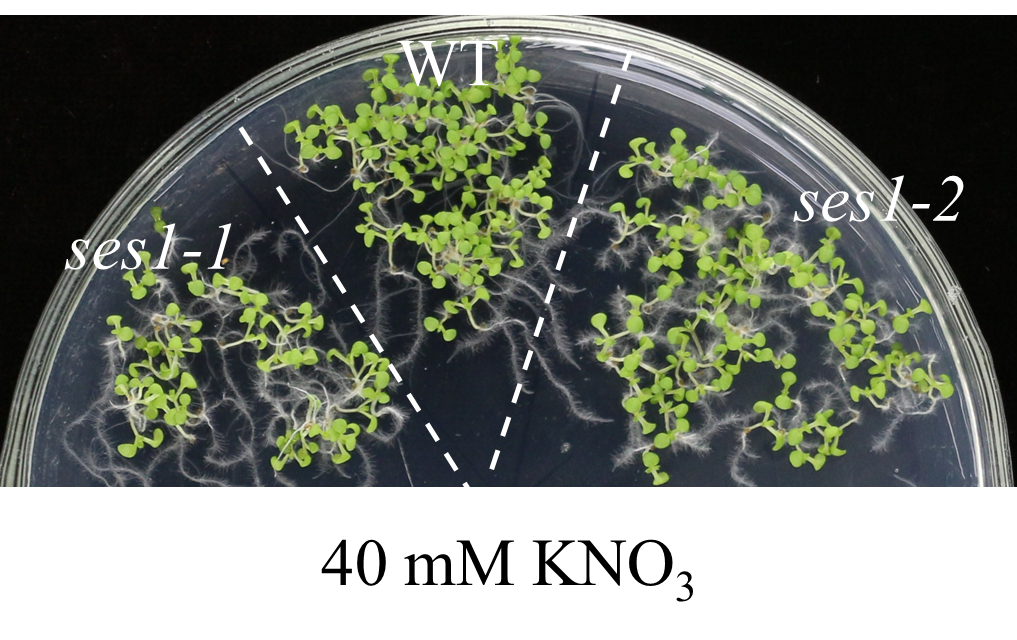

Supplement: Supplemental Information 1 [file peerj-10-14282-s001.zip › raw data1/Figure 2/Figure 2A/40mM KNO3.png]

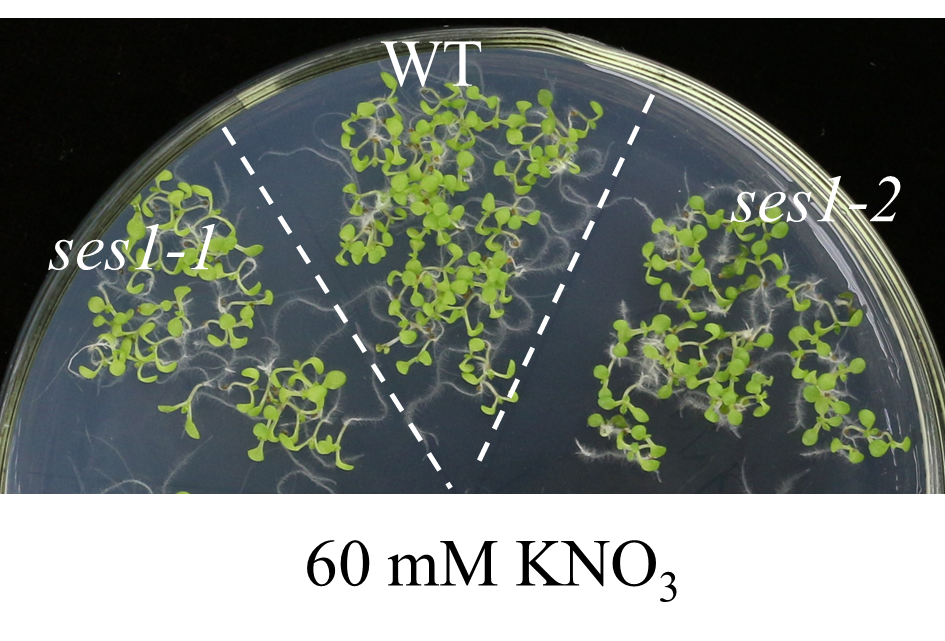

Supplement: Supplemental Information 1 [file peerj-10-14282-s001.zip › raw data1/Figure 2/Figure 2A/60mM KNO3.png]

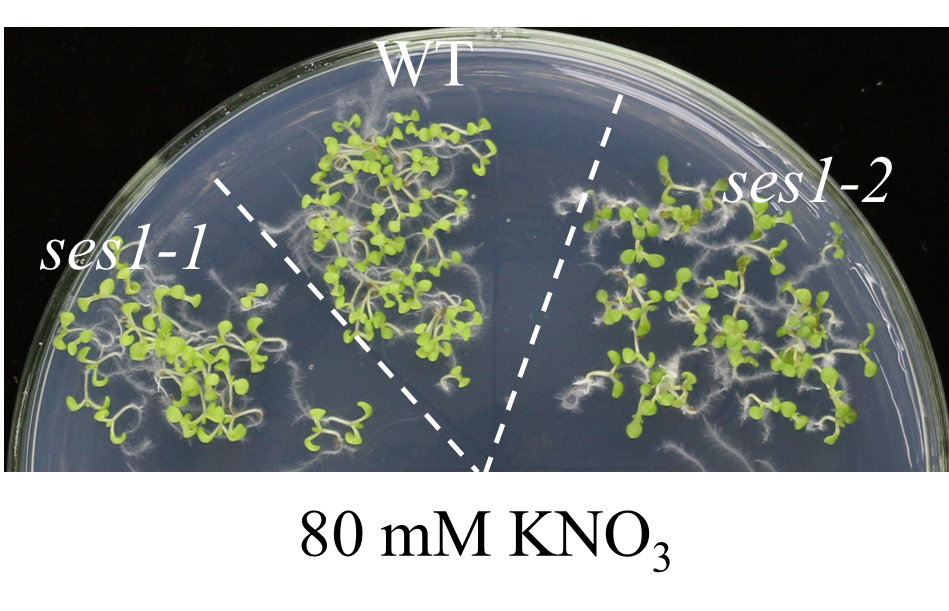

Supplement: Supplemental Information 1 [file peerj-10-14282-s001.zip › raw data1/Figure 2/Figure 2A/80mM KNO3.png]

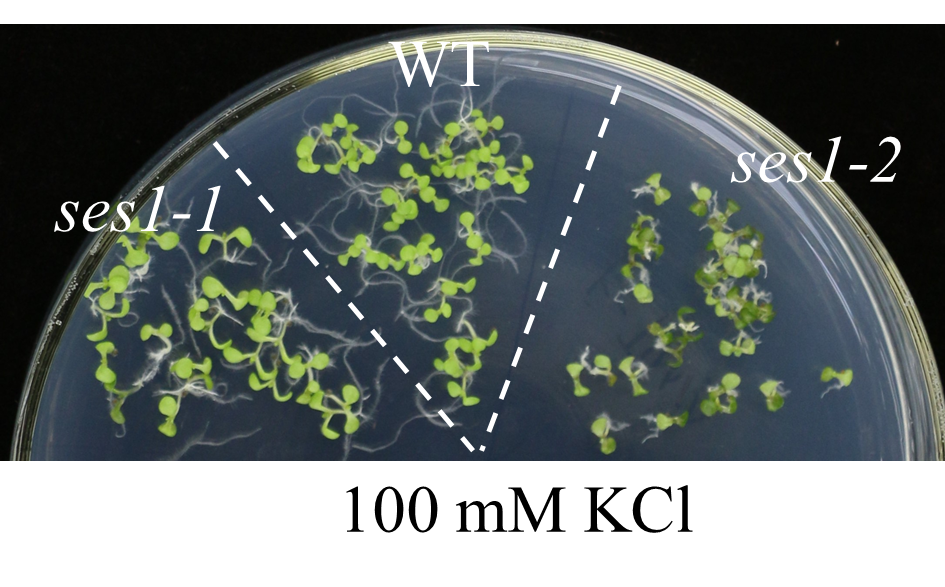

Supplement: Supplemental Information 1 [file peerj-10-14282-s001.zip › raw data1/Figure 2/Figure 2B/100mM KCl.png]

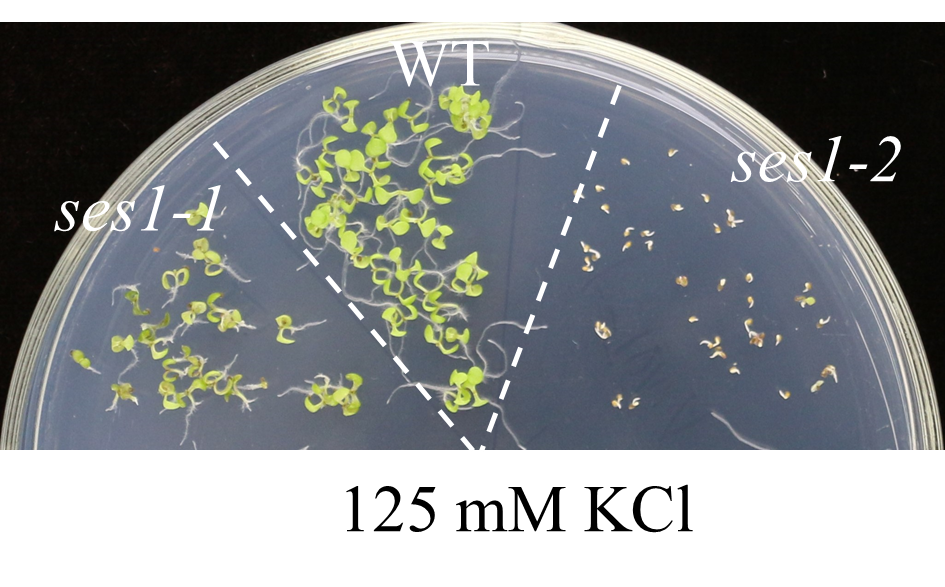

Supplement: Supplemental Information 1 [file peerj-10-14282-s001.zip › raw data1/Figure 2/Figure 2B/125mM KCl.png]

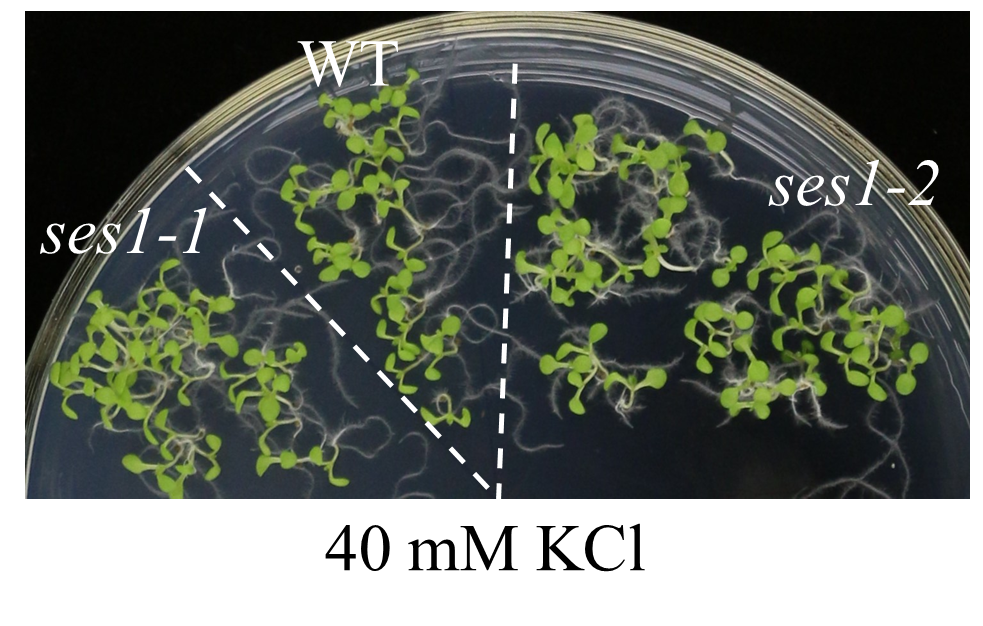

Supplement: Supplemental Information 1 [file peerj-10-14282-s001.zip › raw data1/Figure 2/Figure 2B/40mM KCl.png]

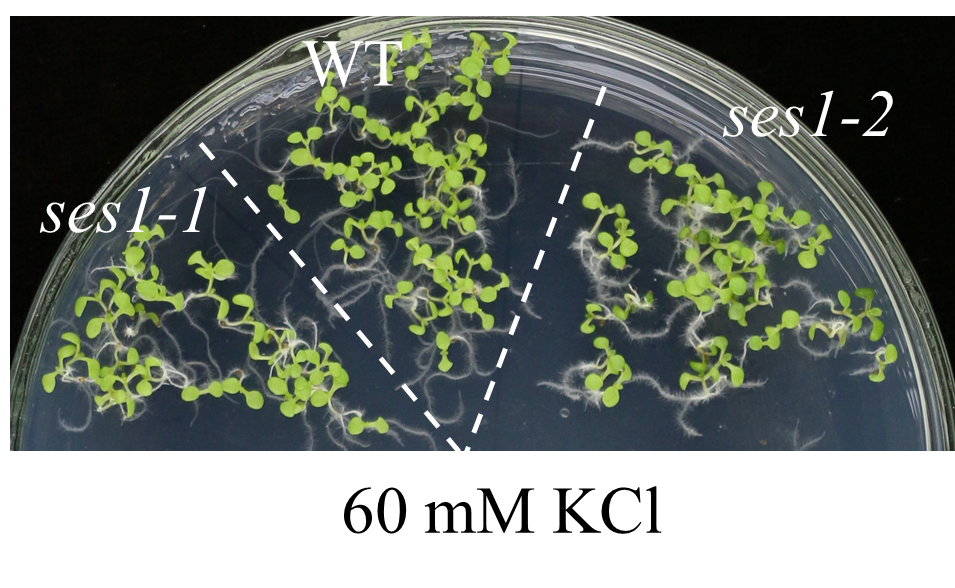

Supplement: Supplemental Information 1 [file peerj-10-14282-s001.zip › raw data1/Figure 2/Figure 2B/60mM KCl.png]

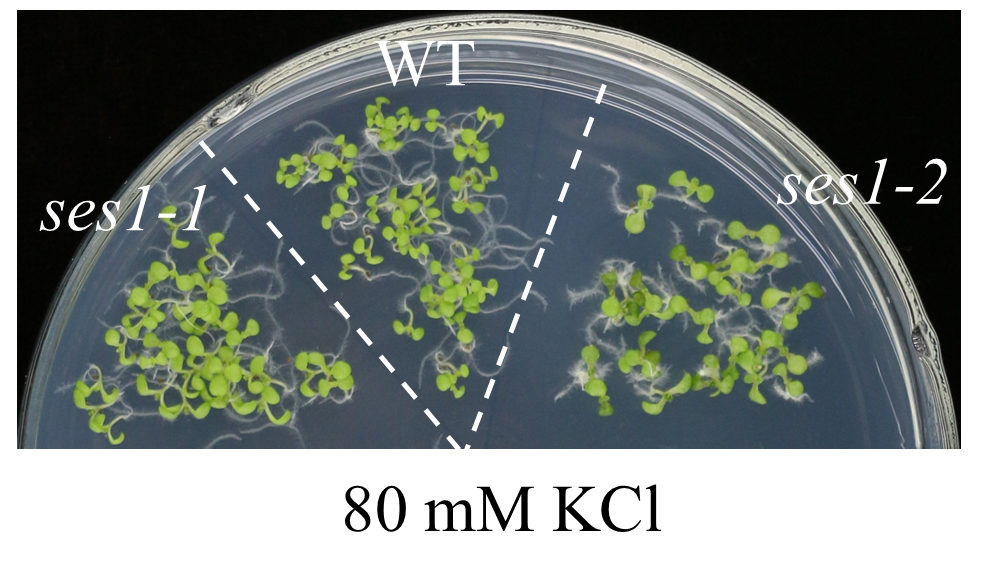

Supplement: Supplemental Information 1 [file peerj-10-14282-s001.zip › raw data1/Figure 2/Figure 2B/80mM KCl.png]

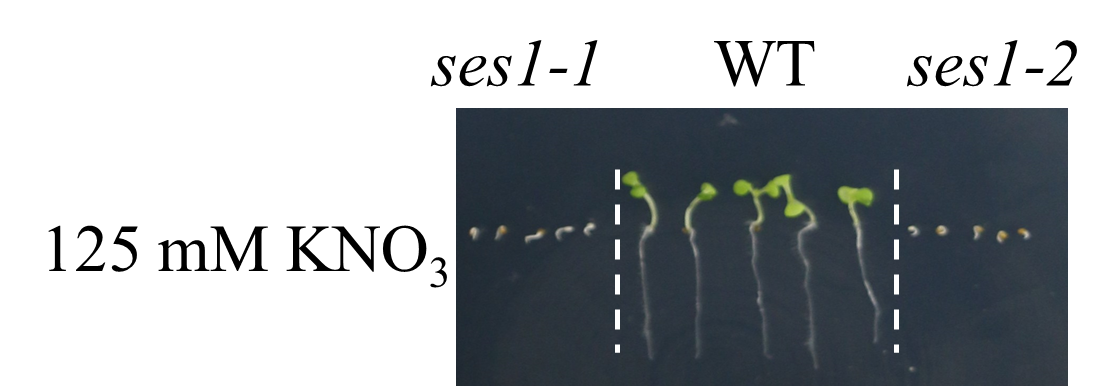

Supplement: Supplemental Information 1 [file peerj-10-14282-s001.zip › raw data1/Figure 2/Figure 2C/125mM KNO3.png]

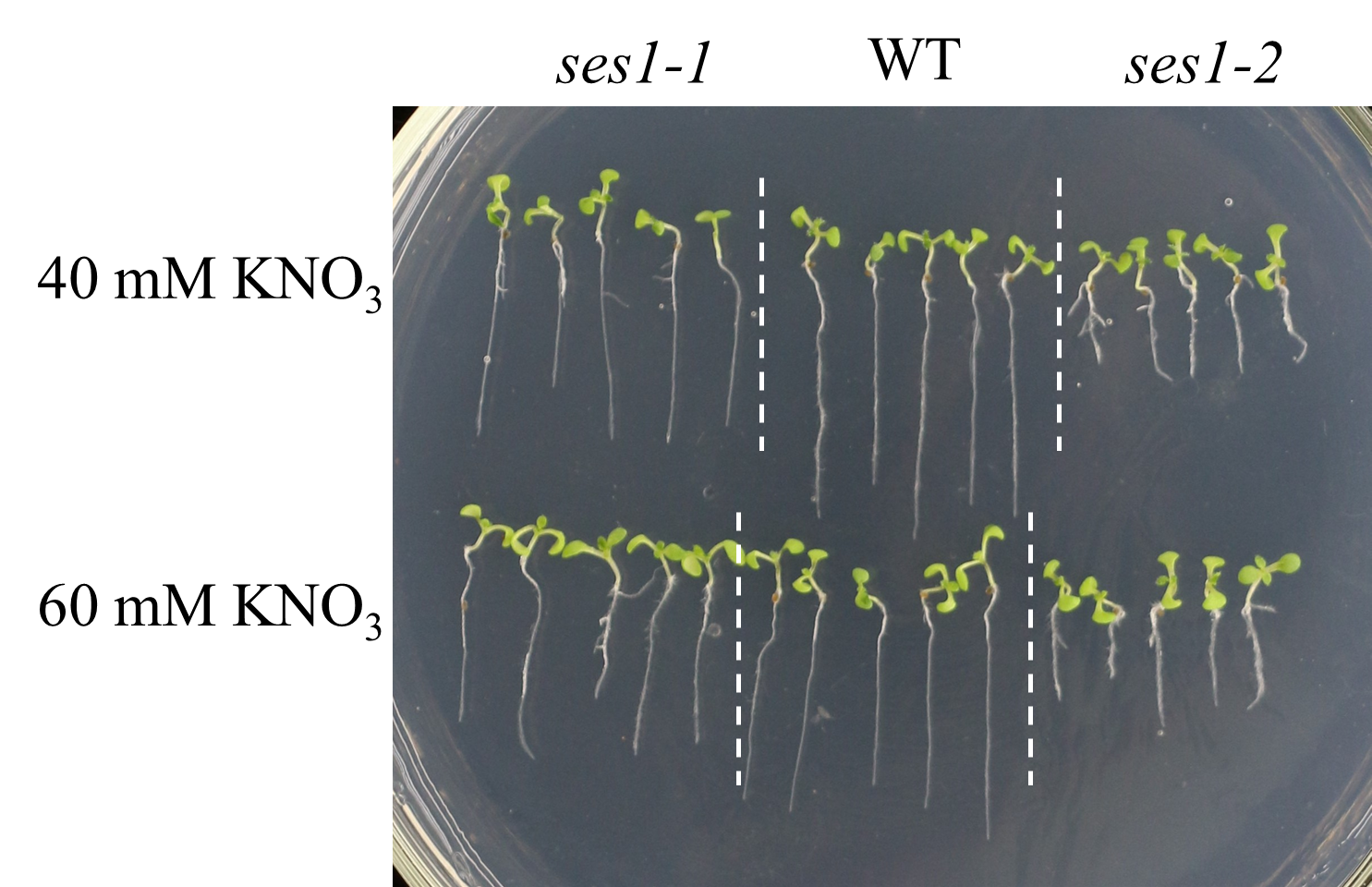

Supplement: Supplemental Information 1 [file peerj-10-14282-s001.zip › raw data1/Figure 2/Figure 2C/40mM+60mM KNO3.png]

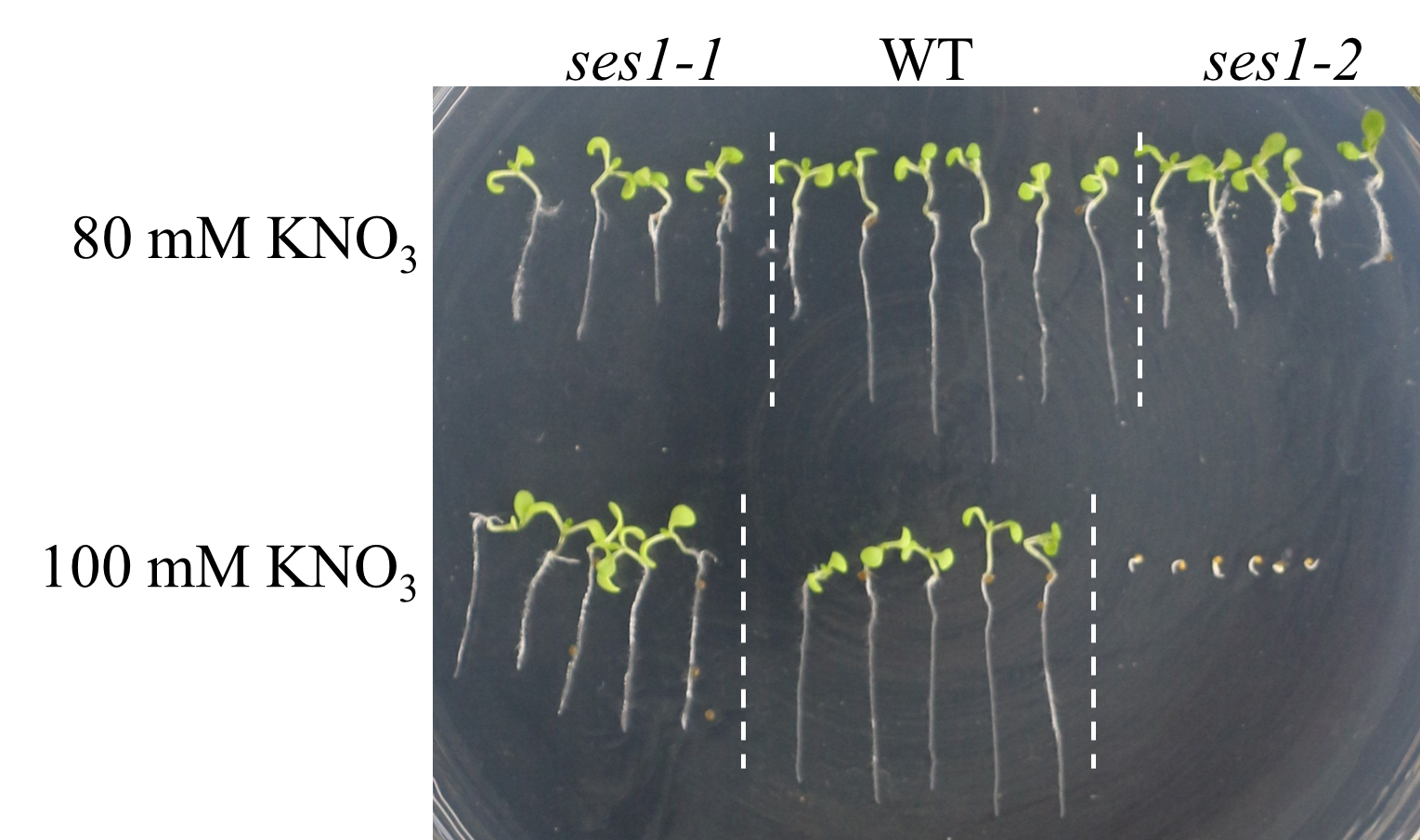

Supplement: Supplemental Information 1 [file peerj-10-14282-s001.zip › raw data1/Figure 2/Figure 2C/80mM+100mM KNO3.png]

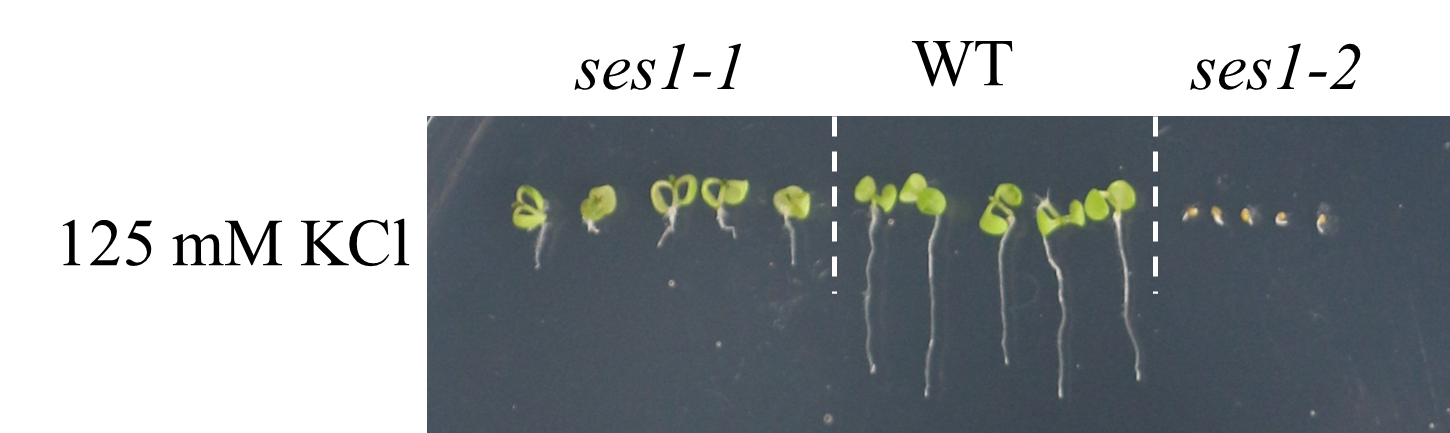

Supplement: Supplemental Information 1 [file peerj-10-14282-s001.zip › raw data1/Figure 2/Figure 2D/125mM KCl.png]

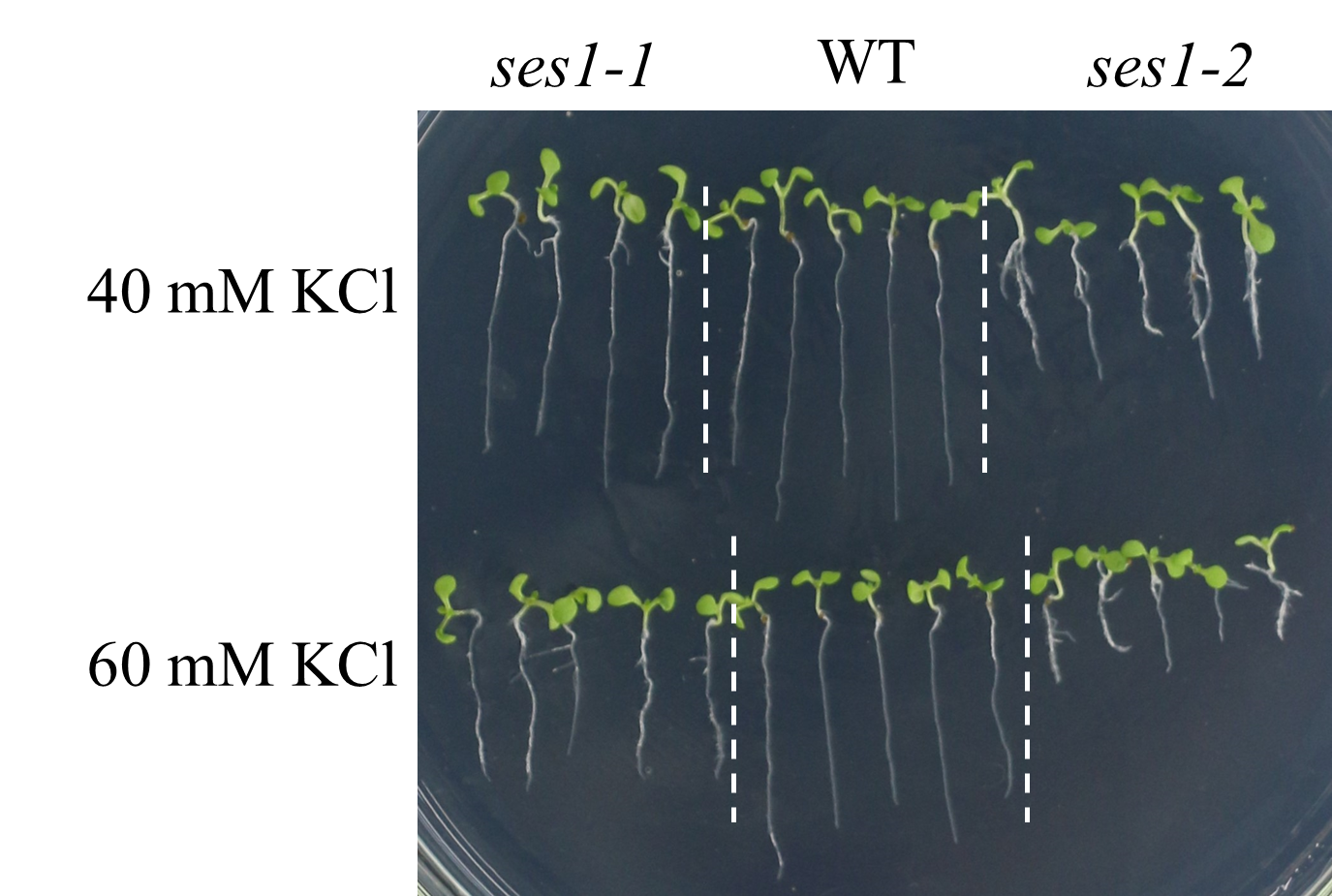

Supplement: Supplemental Information 1 [file peerj-10-14282-s001.zip › raw data1/Figure 2/Figure 2D/40mM+60mM KCl.png]

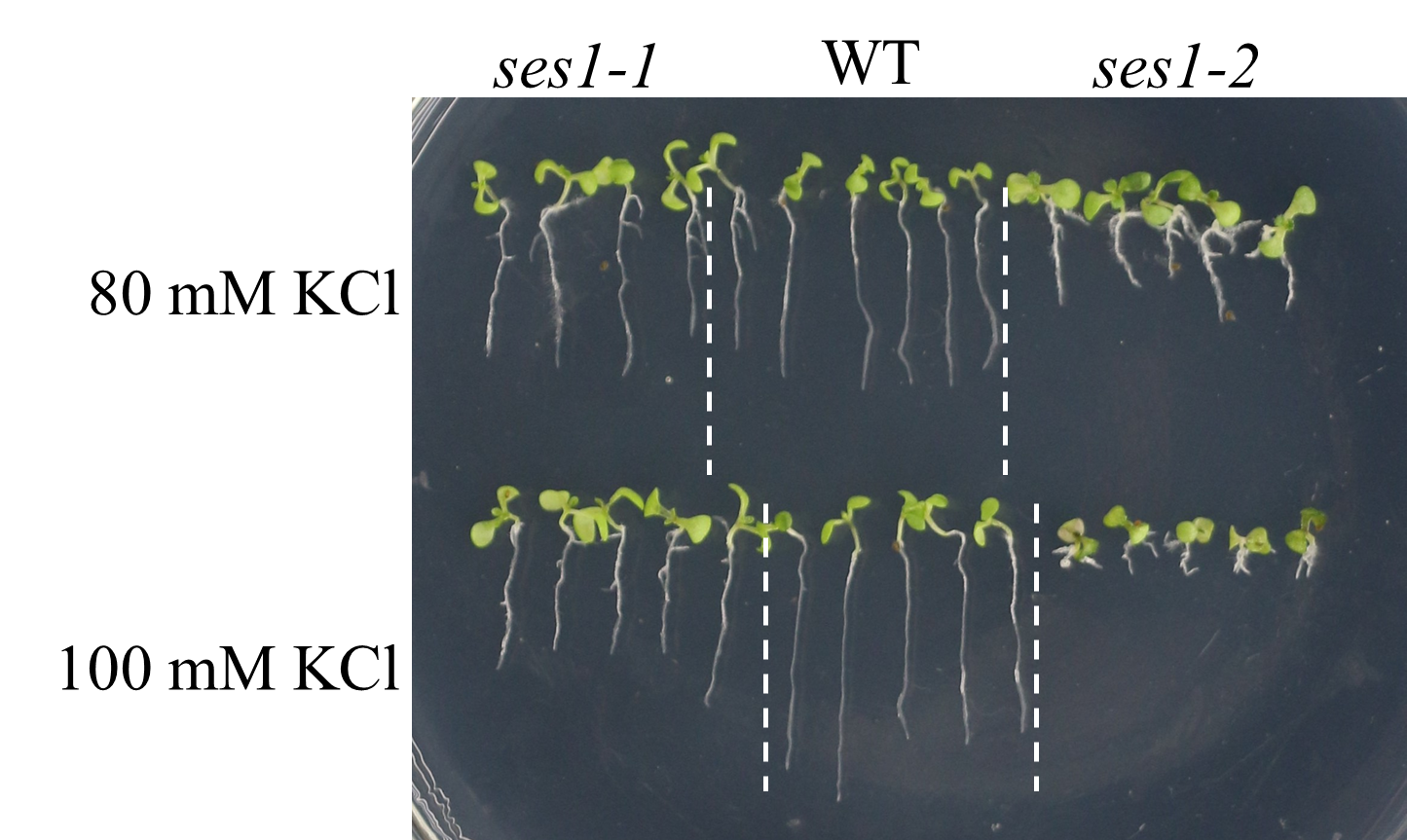

Supplement: Supplemental Information 1 [file peerj-10-14282-s001.zip › raw data1/Figure 2/Figure 2D/80mM+100mM KCl.png]

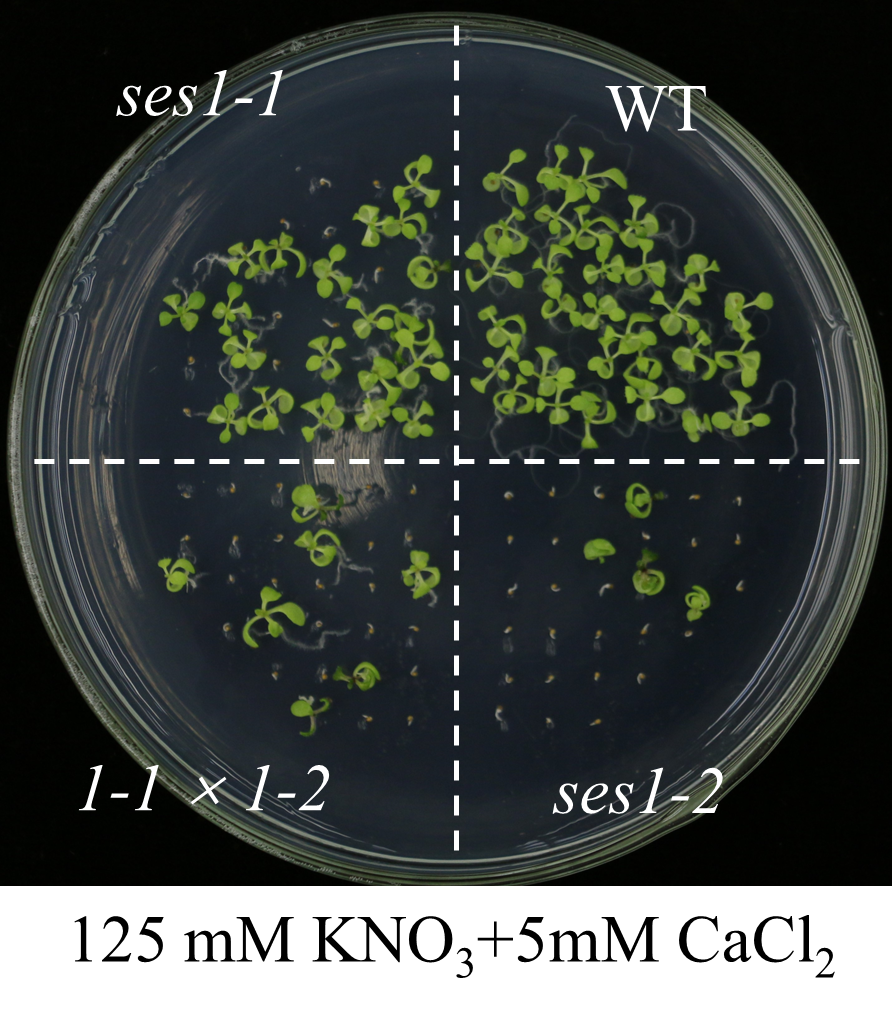

Supplement: Supplemental Information 2 [file peerj-10-14282-s002.zip › raw data2/Figure 3/Figure 3A/125 mM KNO3+5mM CaCl2.png]

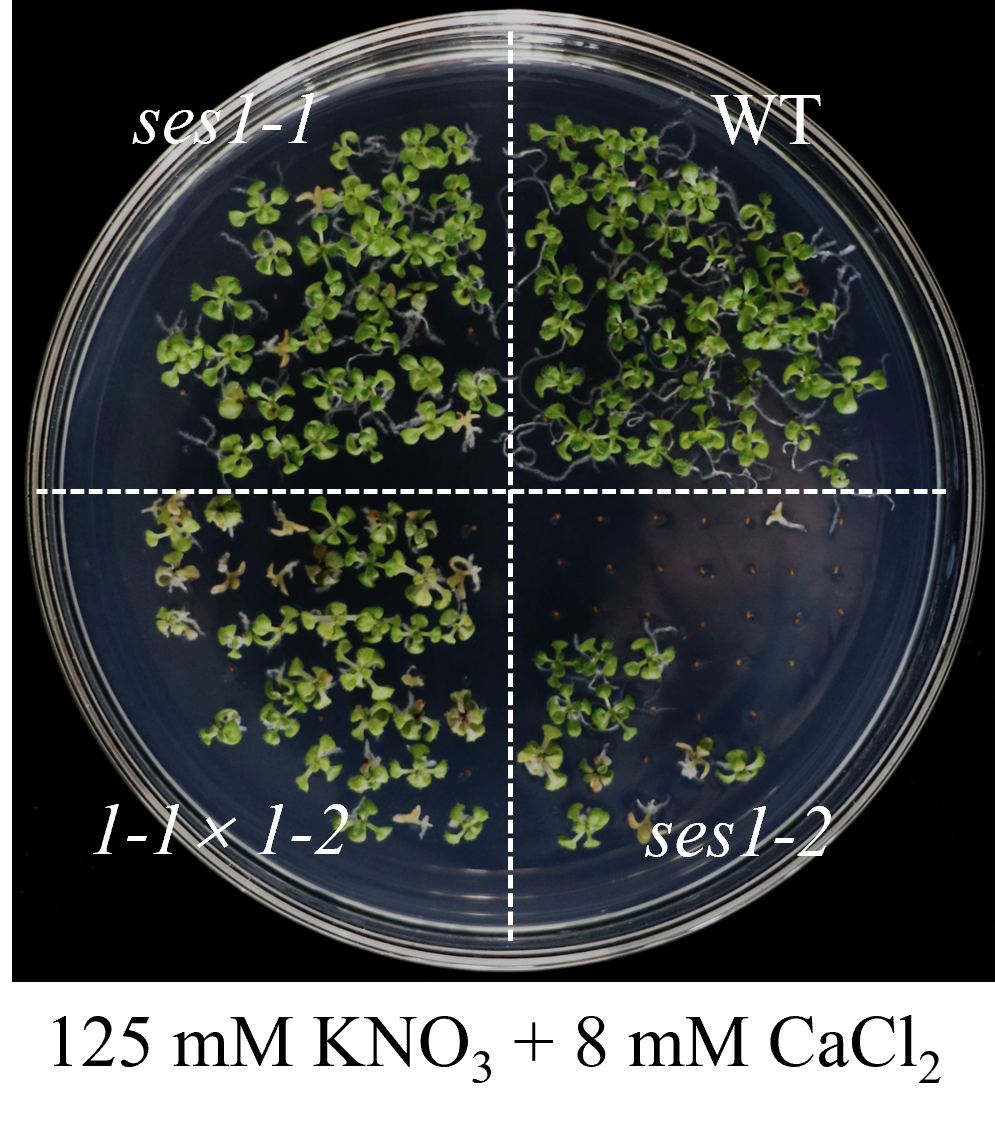

Supplement: Supplemental Information 2 [file peerj-10-14282-s002.zip › raw data2/Figure 3/Figure 3A/125 mM KNO3+8mM CaCl2.png]

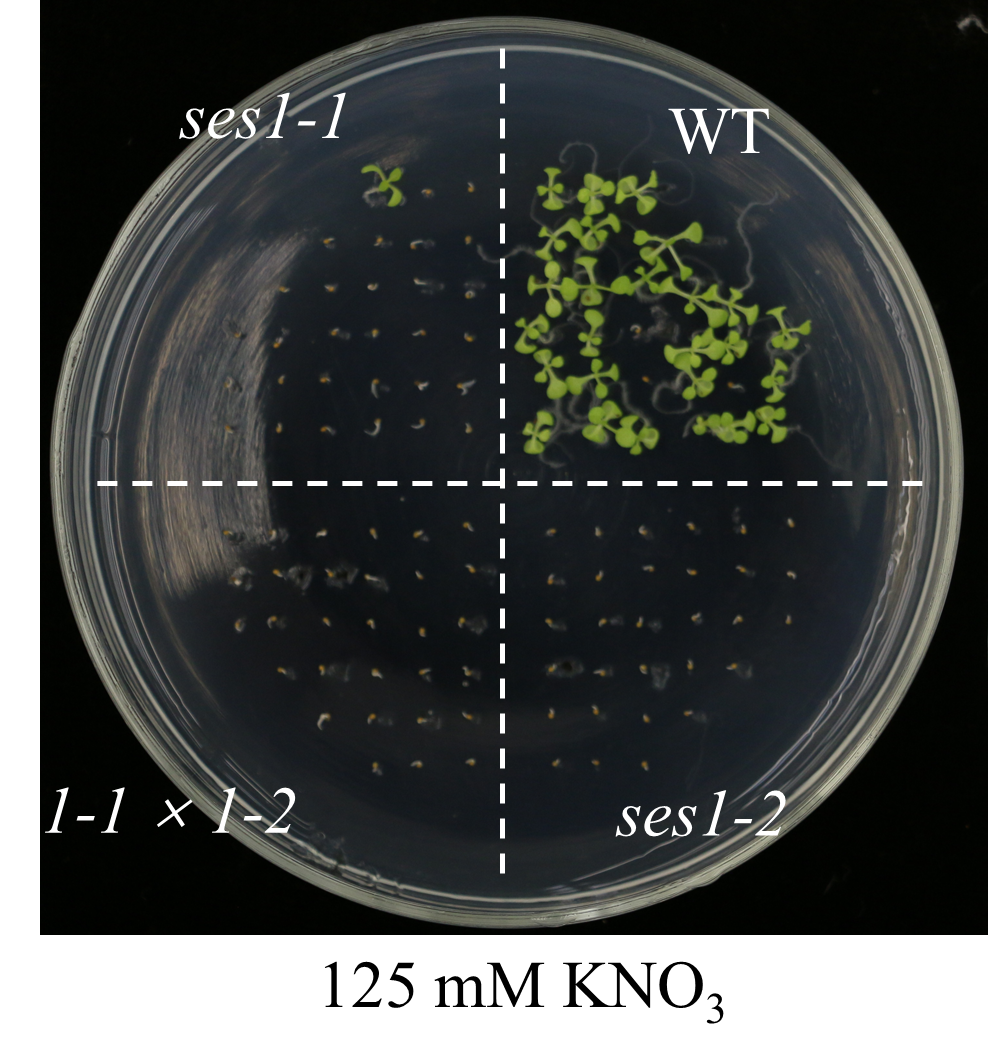

Supplement: Supplemental Information 2 [file peerj-10-14282-s002.zip › raw data2/Figure 3/Figure 3A/125 mM KNO3.png]

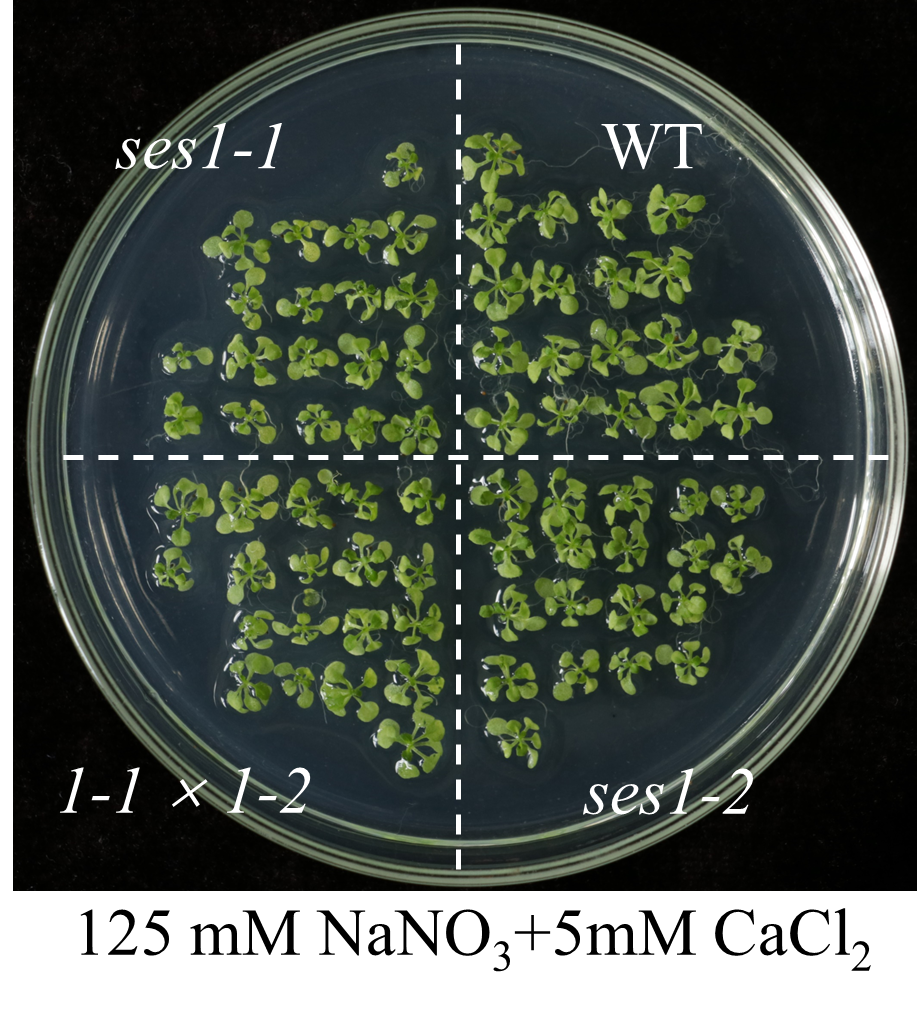

Supplement: Supplemental Information 2 [file peerj-10-14282-s002.zip › raw data2/Figure 3/Figure 3A/125 mM NaNO3+5mM CaCl2.png]

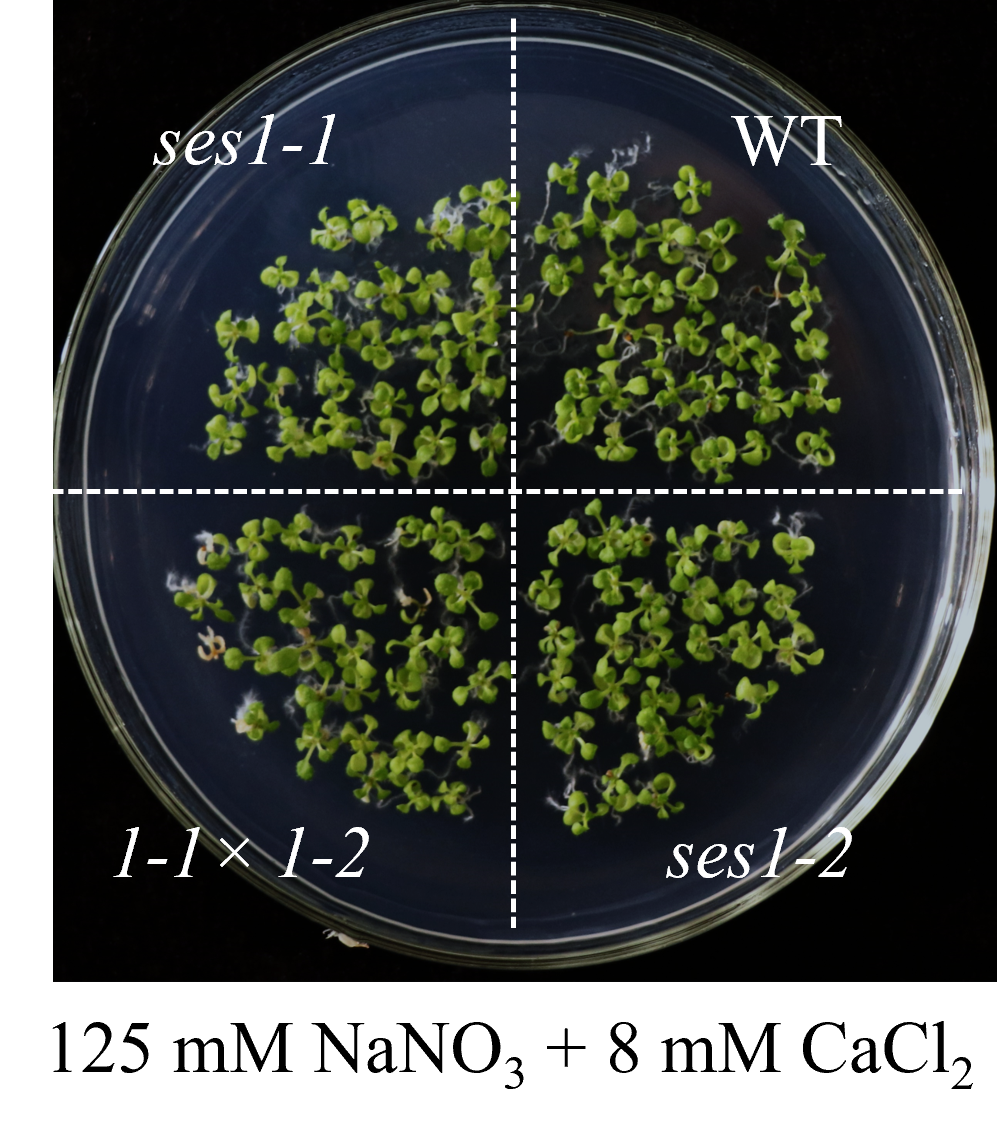

Supplement: Supplemental Information 2 [file peerj-10-14282-s002.zip › raw data2/Figure 3/Figure 3A/125 mM NaNO3+8mM CaCl2.png]

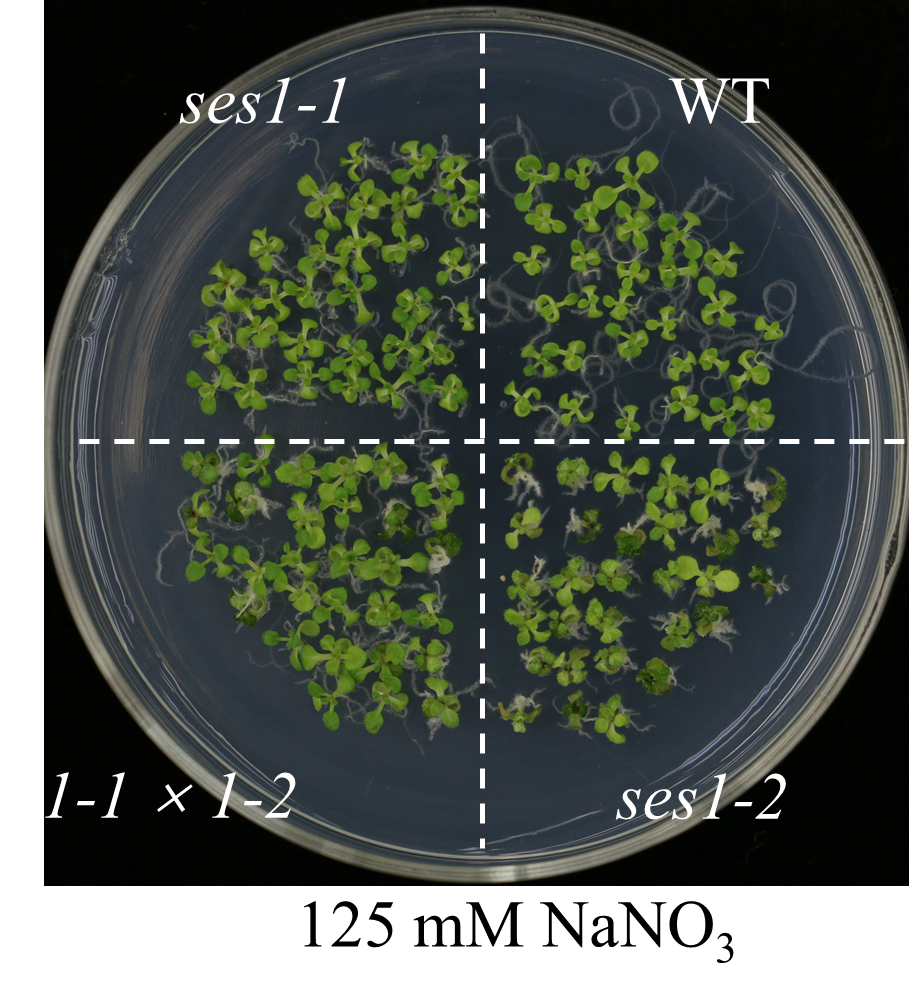

Supplement: Supplemental Information 2 [file peerj-10-14282-s002.zip › raw data2/Figure 3/Figure 3A/125 mM NaNO3.png]
