# Supplementary material for: SES1 is vital for seedling establishment and post-germination growth under high-potassium stress conditions in Arabidopsis thaliana
Source: PeerJ. 2022 Oct 31;10:e14282. doi: 10.7717/peerj.14282 (PMC9632470; doi:10.7717/peerj.14282)
Supplement: Supplemental Information 3 [file peerj-10-14282-s003.docx]

**Table S1. Primers used in this study.**

Primer name Primer sequence (5′-3′)

*UBQ10*-F 5′-GCCAAGATCCAGGACAAGG-3′

*UBQ10*-R 5′-CGCAGGACCAAGTGAAGAG-3′

*Actin7*-F 5′-GGTCGTACAACCGGTATTGT-3′

*Actin7*-R 5′-GAAGAGCATACCCCTCGTA-3′

*AKT2*-F 5′-TCTTCTTCCTCCTTCACTTG-3′

*AKT2*-R 5′-CATCGTCTCACTTCCATCA-3′

*KAT1*-F 5′-CAACAGTCAATAGCAATAGAT-3′

*KAT1*-R 5′-AAGTCGGATTCGTAACAT-3′

*KAT2*-F 5′-TAATCCTTCCTGCTTCCA-3′

*KAT2*-R 5′-TCTAATCACATCTTCATCATCTAT-3′

*KAT3*-F 5′-CTTCTTATATCATCGGTATCAT-3′

*KAT3*-R 5′-TGTTCTTGCTTGTGTATC-3′

*KUP2*-F 5′-GATCAACATCGTCAGCAGAA-3′

*KUP2*-R 5′-TCCTCTACAGTTCCTTCTCA-3′

*KUP3*-F 5′-TTGAAGAAGATGGCGATA-3′

*KUP3*-R 5′-ATGTGAGGAATGTTGAGT-3′

*KUP4* -F 5′-TTGTGACTACTTGGTTGATG-3′

*KUP4* -R 5′-GATGAAGAGGACGGAGAA-3′

*KCO5*-F 5′-TCATCAACACCATCATCATCATA-3′

*KCO5*-R 5′-GACGACGCATAACGCTAA-3′

*SKOR*-F 5′-CATTCTTCGTTGCTTATC-3′

*SKOR*-R 5′-AGGTATCTCACTTCTTCT-3′

*GORK*-F 5′-GACATTACATTATTCCTTATTCAG-3′

*GORK*-R 5′-AACCAATCACTCCTTCTT-3′

*BiP1*-F 5′-GTGGCGAGAAGAATATCCT-3′

*BiP1*-R 5′-TCCGTTATCAATGGTCAAGA-3′

*BiP3*-F 5′-GTGGTTCGTATAATCAAT-3′

*BiP3*-R 5′-TTGTCTATGGTAAGGATA-3′

*CNX*-F 5′-TGCCTACAAGTCTAAGAT-3′

*CNX*-R 5′-AGAAGAATACAACCACAAT-3′

*DER1*-F 5′-AGTCCTTCATCCTCTTGCTA-3′

*DER1*-R 5′-CGCCATCTTGCTACGATT-3′

*ERO1*-F 5′-TAGATAACAGAGCATTCA-3′

*ERO1*-R 5′-GTCCAGTATAACCAGTAT-3′

*HRD1*-F 5′-AGAGTGGAGTATATTGAGA-3′

*HRD1-*R 5′-GAAGAAGACTGACATTGA-3′

*IRE1A*-F 5′-CAAGAGCAACATAAGCAGAA-3′

*IRE1A*-R 5′-AACAGAGACAACAACACCTA-3′

*SEL1*-F 5′-AGGTAGAGACTTGGATAGAGAA-3′

*SEL1*-R 5′-GGAGATAGAGAATTGTGATGAGA-3′
